# Sleep endoscopy as a complementary diagnostic method for snoring and sleep apnea

**DOI:** 10.31744/einstein_journal/2022MD8035

**Published:** 2022-08-15

**Authors:** Dalila Araújo Mota, Marcelo Gervilla Gregorio, Altair da Silva Costa, Denilson Stork Fomin, Marcia Jacomelli

**Affiliations:** 1 Hospital Israelita Albert Einstein São Paulo SP Brazil Hospital Israelita Albert Einstein, São Paulo, SP, Brazil.; 2 Escola Paulista de Medicina Universidade Federal de São Paulo São Paulo SP Brazil Escola Paulista de Medicina, Universidade Federal de São Paulo, São Paulo, SP, Brazil.

**Keywords:** Sleep apnea, obstructive/diagnosis, Snoring, Endoscopy/methods, Airway obstruction

## Abstract

Obstructive sleep apnea is a disorder characterized by complete or incomplete and recurrent upper airway collapse induced by sleep. Several diagnostic methods for obstructive sleep apnea are used, but only sleep endoscopy allows an endoscopic assessment of pharyngeal collapse during sedation. It is essential to carry out sleep endoscopy following a systematic institutional protocol, in preestablished stages, to ensure better reproducibility and reliability of the results found. Sleep endoscopy has few limitations and is a safe test, with a low risk of complications.

## INTRODUCTION

Obstructive sleep apnea is a common disorder that affects 4% of middle-aged men and 2% of women. Nowadays, it has an even higher prevalence, reaching 23% in women and 50% in men. It is characterized by complete or incomplete and recurrent collapse of the upper airways during sleep, which leads to hypoxemia and hypercapnia, with consequent sleep fragmentation and awakening to restore the airway patency.^(1,2)^

The current gold standard diagnostic method for obstructive sleep apnea is polysomnography. There are several methods for the topographic diagnosis of upper airway obstruction in patients with obstructive sleep apnea. For the static (anatomical) evaluation, physical and imaging examinations are available, such as computed tomography of the face and neck. For the dynamic evaluation, there are endoscopic exams, such as nasofibroscopy and drug induced sleep endoscopy (DISE), also called sleep endoscopy.^(2,3)^ The initial clinical investigation of the patient is carried out through history taking and a standard otorhinolaryngological examination, using nasofibroscopy and the Müller maneuver during the exam. This allows both the static and dynamic assessment of upper airway collapse, and has a good correlation with the topography of the velopharynx alone. However, since this test is performed with the patient awake and in a sitting position, it has been shown to be ineffective as a predictor of upper airway obstruction.^(2)^ Likewise, computed tomography is performed with the patient awake and lying down and, despite allowing three-dimensional anatomical assessment of the upper airways, it does not allow assessment of a pharyngeal lumen obstruction during sleep.^(3)^

Therefore, DISE is the only method available that allows both static and dynamic assessment of the upper airway during drug-induced sleep. It is performed under sedation and with direct visualization of the pharynx, allowing a better assessment of the upper airway patency. This method was designed as a result of the necessity for a better assessment of pharyngeal collapse during sleep, as a result of the high failure rates of the proposed surgical treatments, since the clinical evaluation and the imaging examinations alone, performed during wakefulness, were insufficient to predict the exact degree and site of the upper airway collapse that occurs during sleep.^(2,4)^

During the examination, we used a specific and systematized protocol from the *Hospital Israelita Albert Einstein* (HIAE) to provide a better assessment of pharyngeal collapse, and the different forms of individualized treatment, surgical or non-surgical. Importantly, DISE does not replace polysomnography for diagnosis of obstructive sleep apnea.^(5)^

The main indications of DISE in patients with a diagnosis of obstructive sleep apnea are preoperative evaluation of candidates for surgical treatment, patients who had a failed surgical outcome, candidates for treatment with intraoral appliances, and patients who failed treatment with continuous positive airway pressure (CPAP).^(6,7)^

For proper performance of the exam, it is required that all patients prepare in advance, fasting for at least 8 hours, and have a caregiver of legal age. Initially, a difficult airway assessment risk is performed. The patient is then laid down in a position close to the usual one (supine or lateral decubitus), and a venipuncture, cardiovascular monitoring and oximetry are performed. It is essential that the examination is performed in a suitable room, with cardiopulmonary resuscitation and airway management materials. All patients are monitored by the physician who will perform the exam, as well as an anesthesiologist, who is responsible for venous sedation. In addition, the entire exam is documented in photographs and video for further evaluation by the team later on.

**Figure 1:**
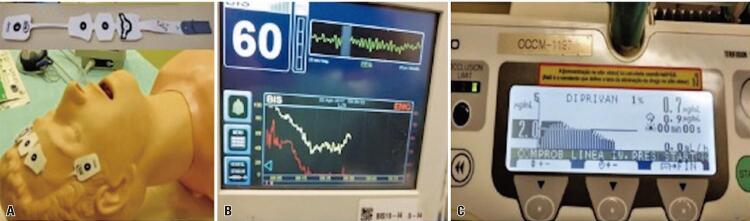
Sedation in sleep endoscopy. (A) Bispectral index electrodes; (B) Bispectral index monitor with numerical representation of the level of sedation and electroencephalographic (yellow) and muscle (red) activity curves; (C) Propofol infusion pump

The first phase of the examination consists of performing an anatomical baseline nasofibrolaryngoscopy for static assessment of the nasopharynx, the oropharynx, and the hypopharynx. After this stage, the induced sleep phase begins, which, in our organization, is performed with propofol, at an approximate dose of 1.50mcg/mL to 3.0mcg/mL. The entire study is dynamic with direct assessment of pharyngeal collapse sites and snoring events during sleep. In all patients, the bispectral index is used to obtain a better control of the adequate level of sedation (Figure 1), because ideally the sedation should not be too deep or superficial, but enough for the patient to experience obstructive events (snoring/apnea).^(8)^

The sedation protocol of the organization preferably recommends the use of a single drug, but other medications, such as dexmedetomidine, may be used, depending on the individualized pre-anesthetic evaluation.^(8)^

To evaluate upper airway collapse, the most used protocol is VOTE (Table 1).^(5)^ The findings are classified by this method, because it seems to be the most understandable, and each letter represents a topography to be evaluated (V: velum, O: oropharynx, T: tongue, and E: epiglottis).^(5)^ In this classification, the severity of obstruction is indicated by the numbers zero, one, and two, depending on the percentage of obstruction.^(6)^ When the narrowing of the assessed region ranges from zero to 50%, the severity of obstruction is classified as zero, or no obstruction; when it ranges from 50% to 75%, it is classified as one, or partial obstruction; and when it is >75%, it is classified as two, or complete obstruction. In the velum region, the narrowing can be anteroposterior, concentric, or lateral. In the oropharynx, it can be lateral or concentric. On the other hand, in the base of the tongue, only anteroposterior narrowing can be found, whereas in the epiglottis, anteroposterior (flap), laterolateral narrowing or epiglottis collapse, resulting from the fall of the tongue, may be observed.^(5)^

**Table 1:** VOTE classification

Obstruction sites	Degree of obstruction^*^	Form of obstruction		
V - velum	0/1/2/x	Concentric	Laterolateral	Anteroposterior
		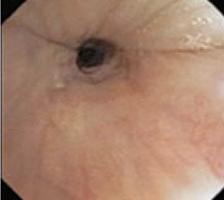	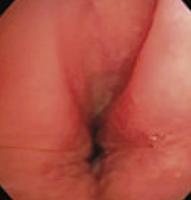	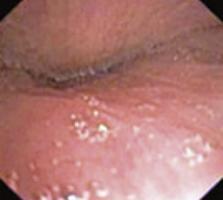
O - oropharynx	0/1/2/x	X	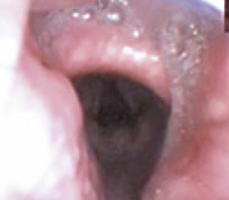	X
T - tongue	0/1/2/x	X	X	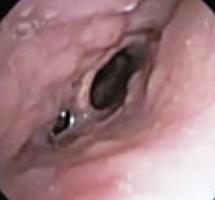
E - epiglottis	0/1/2/x	X	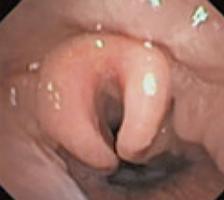	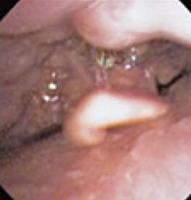

Source: adapted from Hohenhorst W, Ravesloot MJ, Kezirian EJ, de Vries N. Drug-induced sleep endoscopy in adults with sleep-disordered breathing: technique and the VOTE Classification system. Oper Tech Otolaryngol Head Neck Surg. 2012;23(1):11-8.^(9)^

* Degree of obstruction: zero, if up to 50%; one, if 50-75%; two, if >75%; X: not visualized.

In the final stage of the examination, three maneuvers are performed to open the upper airway: slight anteriorization of the mandible, closure of the mouth and lateralization of the head.^(9,10)^ The DISE report must contain a detailed anatomical description, a video recording of the collapse and the grading of the obstruction according to the VOTE protocol, as well as the upper airway opening maneuvers.

## CONCLUSION

Drug induced sleep endoscopy, or sleep endoscopy, is an exam with practical use in the evaluation of pharyngeal collapse in patients with obstructive sleep apnea. It has few limitations and is safe as long as an adequate setting is used and a systematized protocol is carried out by trained professionals.
